# Toll-like receptor 5-mediated signaling enhances liver regeneration in mice

**DOI:** 10.1186/s40779-021-00309-4

**Published:** 2021-02-23

**Authors:** Wen Zhang, Lei Wang, Xue-Hua Sun, Xian Liu, Yang Xiao, Jie Zhang, Ting Wang, Hui Chen, Yi-Qun Zhan, Miao Yu, Chang-Hui Ge, Chang-Yan Li, Guang-Ming Ren, Rong-Hua Yin, Xiao-Ming Yang

**Affiliations:** 1 Department of Pharmaceutical Engineering, School of Chemical Engineering and Technology, Tianjin University, Tianjin, 300072 China; 2 State Key Laboratory of Proteomics, Beijing Proteome Research Center, National Center for Protein Sciences (Beijing), Beijing Institute of Lifeomics, Beijing, 102206 China; 3 School of Basic Medical Sciences, Anhui Medical University, Hefei, 230032 Anhui Province China; 4 Beijing Institute of Radiation Medicine, Beijing, 100850 China

**Keywords:** Liver regeneration, Partial hepatectomy, Toll-like receptor 5, CBLB502, NF-κB

## Abstract

**Background:**

Toll-like receptor 5 (TLR5)-mediated pathways play critical roles in regulating the hepatic immune response and show hepatoprotective effects in mouse models of hepatic diseases. However, the role of TLR5 in experimental models of liver regeneration has not been reported. This study aimed to investigate the role of TLR5 in partial hepatectomy (PHx)-induced liver regeneration.

**Methods:**

We performed 2/3 PHx in wild-type (WT) mice, TLR5 knockout mice, or TLR5 agonist CBLB502 treated mice, as a model of liver regeneration. Bacterial flagellin content was measured with ELISA, and hepatic TLR5 expression was determined with quantitative PCR analyses and flow cytometry. To study the effects of TLR5 on hepatocyte proliferation, we analyzed bromodeoxyuridine (BrdU) incorporation and proliferating cell nuclear antigen (PCNA) expression with immunohistochemistry (IHC) staining. The effects of TLR5 during the priming phase of liver regeneration were examined with quantitative PCR analyses of immediate early gene mRNA levels, and with Western blotting analysis of hepatic NF-κB and STAT3 activation. Cytokine and growth factor production after PHx were detected with real-time PCR and cytometric bead array (CBA) assays. Oil Red O staining and hepatic lipid concentrations were analyzed to examine the effect of TLR5 on hepatic lipid accumulation after PHx.

**Results:**

The bacterial flagellin content in the serum and liver increased, and the hepatic TLR5 expression was significantly up-regulated in WT mice after PHx. TLR5-deficient mice exhibited diminished numbers of BrdU- and PCNA-positive cells, suppressed immediate early gene expression, and decreased cytokine and growth factor production. Moreover, PHx-induced hepatic NF-κB and STAT3 activation was inhibited in *Tlr5*^−/−^ mice, as compared with WT mice. Consistently, the administration of CBLB502 significantly promoted PHx-mediated hepatocyte proliferation, which was correlated with enhanced production of proinflammatory cytokines and the recruitment of macrophages and neutrophils in the liver. Furthermore, *Tlr5*^−/−^ mice displayed significantly lower hepatic lipid concentrations and smaller Oil Red O positive areas than those in control mice after PHx.

**Conclusion:**

We reveal that TLR5 activation contributes to the initial events of liver regeneration after PHx. Our findings demonstrate that TLR5 signaling positively regulates liver regeneration and suggest the potential of TLR5 agonist to promote liver regeneration.

**Supplementary Information:**

The online version contains supplementary material available at 10.1186/s40779-021-00309-4.

## Background

The liver is a unique organ with a strong ability to regenerate itself when a massive loss of hepatic parenchymal cells occurs after hepatic resection [1]. Liver regeneration after partial hepatectomy (PHx) occurs in a multistep process involving at least three important recovery phases: the priming phase, the proliferation phase, and the termination phase [2]. The priming phase is referred to as the immediate early phase of liver regeneration, which occurs very rapidly after PHx and lasts for approximately 4–6 h [2, 3]. The quick induction of immediate early genes, such as *c-fos, c-jun,* and *c-myc*, are hallmarks of this phase [4]. The initiation of liver regeneration is driven by proinflammatory cytokines, especially TNF-α and IL-6, which are secreted mainly from nonparenchymal cells, particularly Kupffer cells (KCs) [2]. After the stimulation of these proinflammatory cytokines, normally quiescent hepatocytes regain proliferative competence and progress through the cell cycle in response to a collection of mitogenic growth factors [5]. After hepatocytes pass the restriction point in G1 in the presence of growth factors, the liver irreversibly enters the proliferative phase [2]. However, the mechanisms through which PHx results in cytokine production and transcription factor activation in the liver remain unclear.

Toll-like receptors (TLRs), which act as innate immune signal sensors and play central roles in host defense, are widely expressed on parenchymal and nonparenchymal liver cells [6, 7]. In addition to their roles in defense against pathogens, TLRs significantly contribute to tissue repair and regeneration [8]. Most TLRs relay signals via MyD88, a common adaptor protein, resulting in the production of various proinflammatory cytokine [8]. MyD88 deletion impairs liver regeneration by attenuating the activation of NF-κB and decreasing immediately early gene expression and TNF-α and IL-6 production in KCs after PHx; these findings highlight that TLRs/MyD88 signaling pathways are crucial for PHx-induced liver regeneration [9, 10]. However, further studies have excluded the possible contributions of TLR2, TLR4, or TLR9 to MyD88-mediated pathways in liver regeneration after PHx in mice [10]. In addition, TLR3 signaling, which uses the distinct adaptor protein TRIF but not MyD88, has been shown to attenuate the initiation of liver regeneration [11]. Therefore, liver regeneration is likely to be driven by some other TLRs via the MyD88 signaling pathway.

TLR5 is the main receptor for bacterial flagellin, and it plays critical roles in regulating the response to ionizing radiation [12, 13] and the immune response [14–16]. CBLB502, a pharmacologically optimized flagellin derivative, has been reported to protect mice against lethal total-body irradiation-induced gastrointestinal and hematopoietic acute radiation syndromes [12], as well as ionizing radiation-induced male reproductive system damage [13]. In recent years, the liver has been identified as one of the most sensitive organs to CBLB502. Administration of CBLB502 strongly activates NF-κB-, STAT3-, AP1-, and PREM-driven pathways in the liver, thus inducing numerous immunomodulatory factors and massive recruitment of immune cells [17]. Moreover, TLR5-mediated pathways have been shown to protect mice against anti-Fas antibody- or concanavalin A (Con A)-induced fulminant liver injury by limiting hepatocyte apoptosis or T/NKT cell activity [17, 18]. Furthermore, studies from our group and others have indicated that TLR5 signaling regulates hepatic immune cell activation and cytokine production [17–20]. Importantly, injection of CBLB502 alone in wild-type (WT) mice induces a rapid increase in serum or hepatic TNF-α, IL-6, and G-CSF, all of which contribute to liver regeneration [2, 17, 21]. Together, these data suggest that TLR5-mediated pathways may be involved in regulating liver regeneration. To our knowledge, the effects of TLR5-medicated pathways on the experimental models of liver regeneration have not previously been studied. In this article, we aimed to determine the role of TLR5 in PHx-induced liver regeneration.

## Methods

### Animals

*Tlr5*^−/−^ mice on a C57BL/6 background from Jackson Laboratory were kindly provided by Prof. Huimin Yan (Wuhan Institute of Virology, Chinese Academy of Sciences, Wuhan, China). Specific-pathogen-free (SPF) C57BL/6 mice were purchased from Vital River Experimental Animal Company (Beijing, China). Male mice between 8 and 10 weeks of age were used in the study. In all experiments, genetically modified mice were systematically compared with their age- and weight-matched WT littermates. All animals were maintained in a temperature-controlled, specific-pathogen-free room with 12-h light and dark cycle and ad libitum diet (standard laboratory chow and water) in the Experimental Animal Center of the Academy of Military Medical Sciences, according to the National Laboratory Animal guidelines (Ministry of Health, China, 1998).

### Partial hepatectomy model

For 2/3 PHx studies, male mice between 8 and 10 weeks of age were anesthetized with 1% pentobarbital sodium. A midline laparotomy was performed by aseptic removal of the central and left lobes [22]. Animals in the control group underwent midventral laparotomy without manipulation of the liver (sham surgery). Age- and weight-matched *Tlr5*^−/−^ mice and WT littermates were treated with gentamycin for 1 week before 2/3 PHx or sham surgery to deplete gram-negative gut-derived bacteria [18]. Experiments were started between 8:00 AM and 12:00 PM. For CBLB502 administration, mice were pretreated with a single dose of CBLB502 (0.2 mg/kg) intraperitoneally 1 h before PHx.

### Gene expression datasets

For gene expression analysis, a published dataset was downloaded from the GEO repository (GSE95135). Raw data were normalized with the robust multiarray average (RMA) method. ExpressVis (http://www.fgvis.com/expressvis/) was used to visualize the expression of specific genes of interest [23].

### Flow cytometry

Liver mononuclear cells (MNCs) were obtained as previously described [18]. Briefly, the liver tissue was dissected and dissociated through collagenase (Sigma-Aldrich, C5138) digestion. Liver specimens were pressed through a 40 μm strainer. The single-cell suspension was centrifuged at 50×*g* for 5 min, and the supernatant was further centrifuged at 320×*g* for 5 min. The pellet containing liver mononuclear cells was resuspended in 10 ml of 30% Percoll (GE, 17089102), and then centrifuged at 800×*g* for 15 min without braking. The cell pellet was washed and then treated with red blood cells lysis solution (TIANGEN Biotech, RT122) and centrifuged at 400×*g* for 10 min. The mononuclear cell pellets were resuspended in RPMI-1640 medium for flow cytometric analysis. MNCs were stained with F4/80-FITC, CD45.2-PE-Cy7, Siglec F-APC, Ly6G-eFluor 450, and CD11b-BV605 to identify liver neutrophils, KCs, and recruited macrophages. TLR5-PE or IgG2a-PE antibody was further used to detect the mean fluorescence intensity (MFI) of TLR5 expression on these cells. After gating of the indicated cells, the Mean of TLR5-PE in the statistics window was used to calculate the TLR5 MFI. The antibodies used are shown in Table S1. The number of MNCs was counted with counting beads. Flow cytometric analysis was performed with a BD FACSCalibur instrument and FlowJo software.

### Histological analysis, oil red O, and immunohistochemistry stainings

Liver tissues were excised and fixed in paraformaldehyde, and then embedded in paraffin. Sections were stained with H&E for morphological analysis. Liver tissues were frozen directly in OCT compound for Oil Red O staining. The liver regeneration rate was determined with PCNA immunohistochemistry staining and the incorporation of bromodeoxyuridine (BrdU). For BrdU incorporation, mice were injected with 85 μg/kg BrdU and sacrificed 2 h later, and incorporation was visualized immunohistochemically with BrdU immunohistochemistry staining. BrdU and PCNA immunohistochemistry staining were performed according to standard protocols (Wuhan Servicebio Technology Co., Ltd.), and the percentage of positive cells was analyzed from three randomly selected fields at 200 × magnification for each sample. Oil Red O positive areas were quantified in three fields per slide under light microscopy (200 ×). All pictures of the liver sections were captured with a Nikon Digital Sight DS-U3 camera. Scale bar, 50 μm. Image analysis procedures were performed with IPP v6.0.

### Measurement of cytokines, flagellin concentrations, and aminotransferases

Serum cytokines were detected with a Mouse TNF Flex Set, Mouse IL-6 Flex Set, Mouse G-CSF Flex Set, Mouse HGF ELISA kit (Abcam, ab223862), and Mouse TGF-α ELISA kit (Cloud-Clone Corp., SEA123Mu) according to the manufacturer’s instructions. A mouse flagellin ELISA kit (Beijing Chengzhi Kewei Biotechnology Co., Ltd., SU-BN28100) was used for quantitative determination of serum and liver homogenate flagellin concentrations. Serum ALT and AST were measured according to the IFCC primary reference procedures at Beijing CIC Clinical Laboratory (Beijing, China).

### Detection of lipids

Serum lipid quantification was performed with a Cholesterol kit (Wako, 294–65,801), Triglyceride kit (Wako, 290–63,701), and NEFA kit (Wako, 294–63,601) according to standard methods. Hepatic lipids were assayed with a Cholesterol kit, Triglyceride kit, and Free fatty acid assay kit.

### Quantitative PCR

Hepatic RNA was extracted with TRIzol and reverse-transcribed into cDNA. The cDNA was amplified with Quantitative PCR Mix. Relative gene expression was evaluated with the ΔCT method, and *Actb* was used as an internal control. Gene specific primers were designed in Primer Bank and are listed in Table S2 in the Supplementary Material.

### Western blotting

Proteins were extracted from liver specimens homogenized with PBS containing proteinase inhibitor cocktail (one tablet for 50 ml, Roche, 04693116001) and 0.5% Triton X-100. Liver total protein quantification was performed with a Protein Quantification Assay Kit. Protein extracts were denatured in Laemmli buffer and then separated through SDS-PAGE. Proteins were transferred from the gel onto a PVDF membrane, which was then probed with the indicated primary antibodies. Immune complexes on the membrane were detected with HRP conjugated secondary antibodies. The antibodies used are shown in Table S1.

### Statistical analysis

Statistical data were calculated in GraphPad Prism 7. Continuous variables are presented as the mean ± standard deviation (SD). Data from two or three independent experiments with similar results are expressed as the mean ± standard error of the mean (SEM). A standard two-tailed unpaired Student’s *t*-test was used to test the significance of differences between the two groups. The distribution of variables was tested with the Kolmogorov–Smirnov test. A *P*-value < 0.05 was considered statistically significant.

## Results

### TLR5 is up-regulated in the liver after PHx

**Fig. 1:**
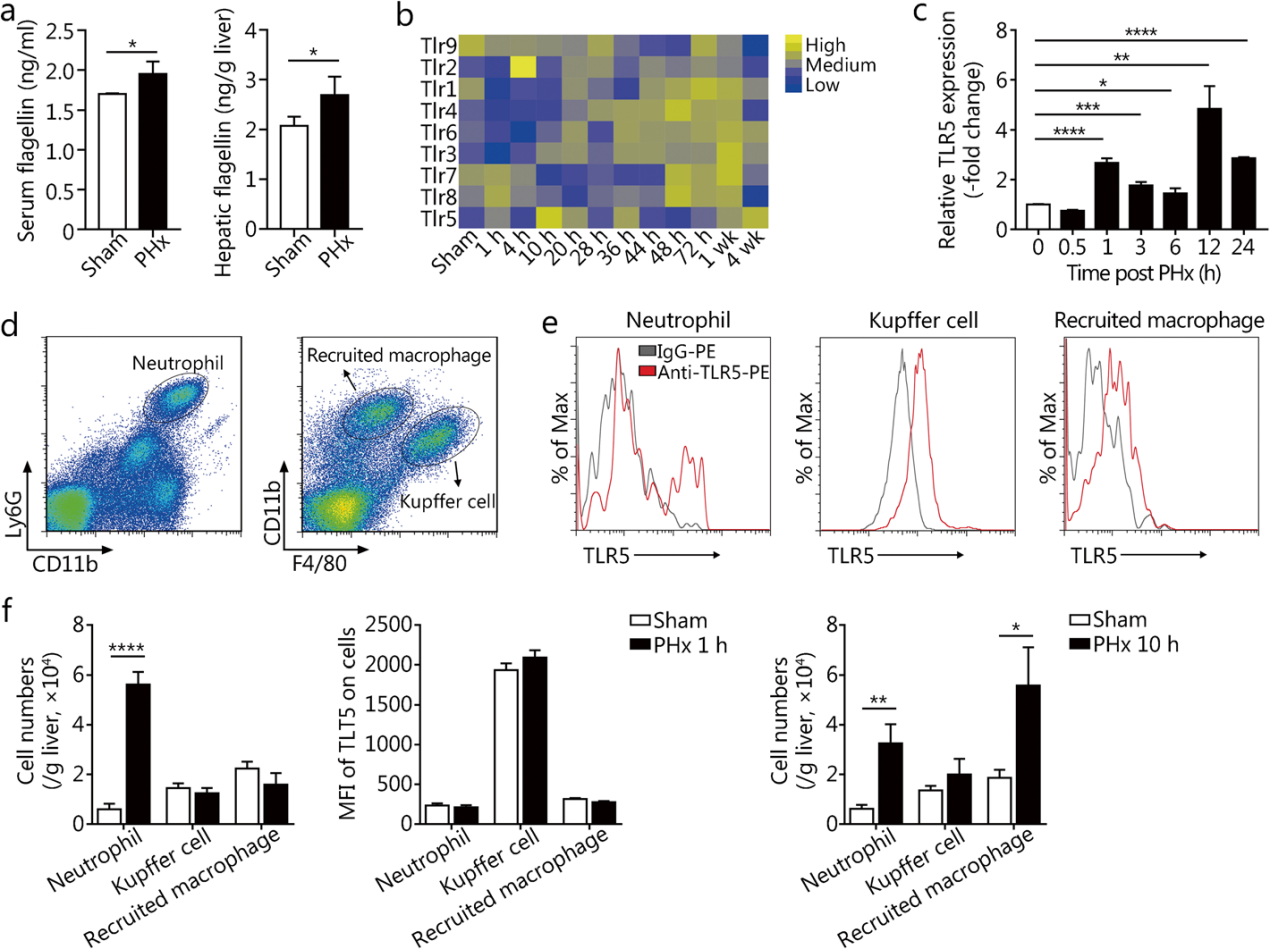
TLR5 is up-regulated in the liver after PHx. **a** Flagellin concentrations in the serum and liver in WT mice 6 h after sham surgery or PHx. **b** TLR expression levels in regenerating WT mouse liver after PHx from a published transcriptome dataset (GSE95135). **c** Relative mRNA levels of hepatic TLR5 at the indicated times after PHx. Flow cytometry gating strategy (**d**) and TLR5 expression levels (**e**) of liver neutrophils, KCs, and recruited macrophages in WT mice. **f** Cell counts and mean fluorescence intensity of TLR5 on liver neutrophils, KCs, and recruited macrophages at the indicated times after PHx. Panel **a** shows means ± SD. Data are representative of three independent experiments. Panels **c** and **f** show means ± SEM. Data are derived from two or three independent experiments with similar results. *n* = 3–6. *P < 0.05, **P < 0.01, ***P < 0.001, ****P < 0.0001

We used 2/3 PHx as an established model of liver regeneration to determine the effect of TLR5 [22]. Significant increases in bacterial flagellin in the serum and liver were observed at 6 h post PHx (Fig. 1a). We next investigated the expression pattern of TLR5 in the liver of mice after PHx. A global transcriptome analysis of the mouse liver at various time points after PHx revealed significant up-regulation of TLR5 along with other TLRs (GSE95135) [24] (Fig. 1b). Consistent with this finding, real-time PCR analysis showed that the expression of TLR5 in the liver significantly increased after PHx, with two peaks occurring at 1 h and 12 h after PHx (Fig. 1c). Flow cytometry indicated that TLR5 was expressed on hepatic neutrophils, KCs, and recruited macrophages (Fig. 1d-e), in line with previous results from our group and others [18, 25]. The mean fluorescence intensity of TLR5 on these cells was unchanged, but the numbers of neutrophils and recruited macrophages significantly increased after PHx, thus potentially accounting for the up-regulation of hepatic TLR5 expression in the early phase after PHx (Fig. 1f).

### TLR5 deficiency decreases hepatocyte proliferation after PHx

**Fig. 2:**
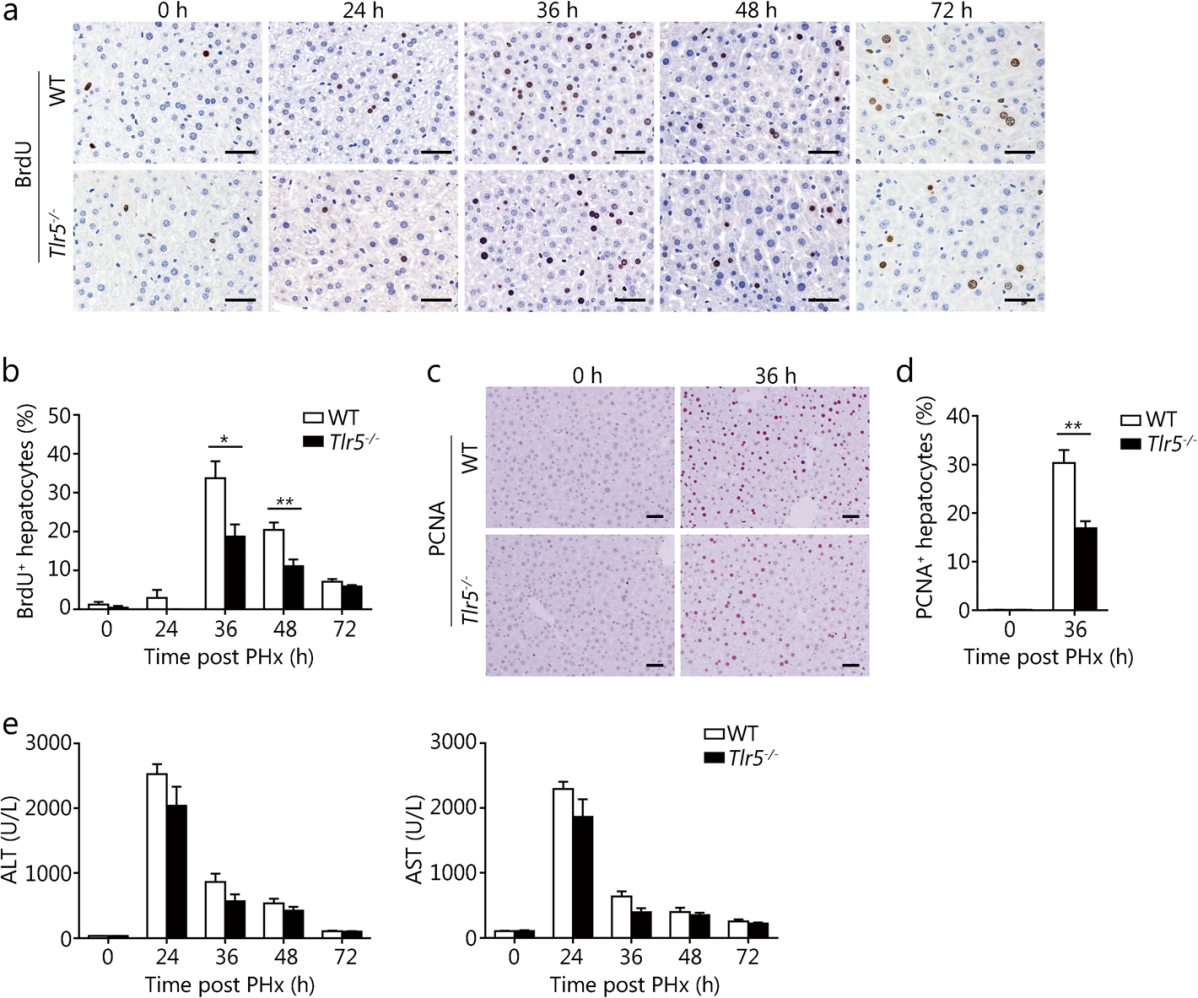
TLR5 deficiency attenuates hepatocyte proliferation after PHx. Proliferation was measured with BrdU and PCNA immunohistochemistry staining. Representative IHC staining images were shown (**a** and **c**), and the percentages of BrdU-positive cells (**b**) and PCNA-positive cells (**d**) were counted. **e** Serum ALT and AST levels in WT and *Tlr5*^−/−^ mice subjected to PHx. Results are expressed as the mean ± SEM. Data are derived from two or three independent experiments with similar results. *n* = 5–6. *P < 0.05, **P < 0.01

To assess the effect of TLR5 on liver regeneration, we performed 2/3 PHx in *Tlr5*^−/−^ mice and their WT littermates. The hepatocyte proliferation, as assessed through the incorporation of BrdU, was markedly decreased in *Tlr5*^−/−^ mice at 36 and 48 h after PHx (Fig. 2a-b). In agreement with this result, PCNA staining showed a decreased number of proliferating hepatocytes in *Tlr5*^−/−^ mice at 36 h after PHx (Fig. 2c-d). The serum ALT and AST levels were rapidly elevated in mice after PHx, but no significant difference was observed between WT and *Tlr5*^−/−^ mice (Fig. 2e), thus indicating a similar degree of liver injury in the two genotypes. Together, these data suggested that TLR5 deficiency significantly decreases hepatocyte proliferation in the first 48 h after PHx.

### TLR5 deficiency suppresses hepatocyte priming in PHx-induced liver regeneration

**Fig. 3:**
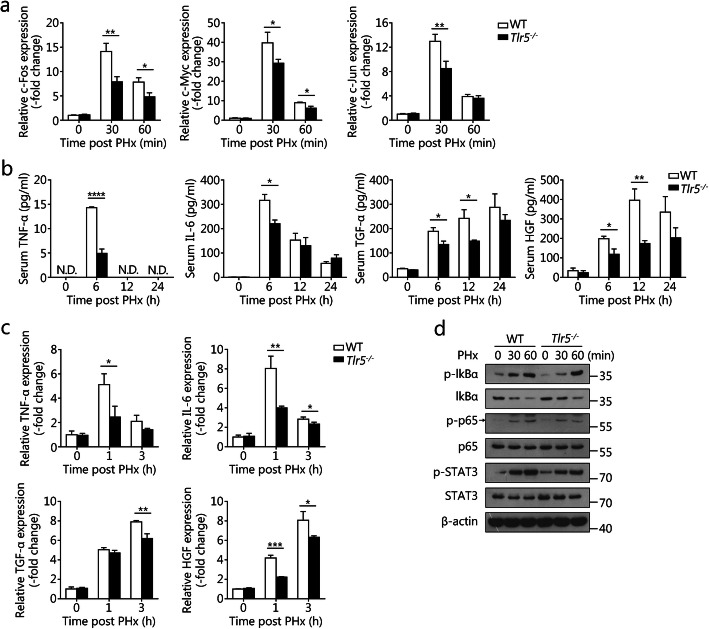
TLR5 deficiency suppresses hepatocyte priming in PHx-induced liver regeneration. **a** Relative mRNA levels of hepatic c-fos, c-myc, and c-jun in WT and *Tlr5*^−/−^ mice at the indicated times after PHx. **b** Serum TNF-α, IL-6, TGF-α, and HGF were measured in regenerating WT and *Tlr5*^−/−^ mouse livers. **c** Quantification of hepatic TNF-α, IL-6, TGF-α, and HGF mRNA expression in WT and *Tlr5*^−/−^ mice at the indicated times after PHx. **d** Western blot analysis of the indicated target proteins in WT and *Tlr5*^−/−^ mouse livers after PHx, with β-actin as the loading control. Panels **a** and **c** show means ± SD. Results are representative of three independent experiments. Panel **b** shows means ± SEM. Data are derived from two or three independent experiments with similar results. *n* = 4–6. Western blot data in panel **d** are representative of three independent experiments. N.D., not detected. *P < 0.05, **P < 0.01, ***P < 0.001, ****P < 0.0001

Because the flagellin concentration and TLR5 expression in the liver increased at early time points after PHx, we examined the effects of TLR5 on the priming of liver regeneration after PHx. Real-time PCR was used to examine gene expression involved in the initial stage of liver regeneration. PHx increased the hepatic mRNA levels of c-Myc, c-Jun, and c-Fos in both *Tlr5*^−/−^ and WT mice at 30 to 60 min after PHx, but the increase was significantly blunted in *Tlr5*^−/−^ mice (Fig. 3a), thus suggesting that loss of TLR5 suppresses PHx-induced immediate early gene expression.

We further investigated the effect of TLR5 signaling on proinflammatory cytokine expression after PHx. As shown in Fig. 3b, the serum levels of TNF-α, IL-6, TGF-α, and HGF in *Tlr5*^−/−^ mice were similar to those in WT mice before PHx. PHx rapidly increased the serum levels of these cytokines in both WT and *Tlr5*^−/−^ mice; however, these effects were greater in WT mice at 6 and 12 h after PHx (Fig. 3b). Consistently with these findings, TNF-α, IL-6, TGF-α, and HGF mRNA levels in the liver were inhibited in *Tlr5*^−/−^ mice at 1 and 3 h after PHx (Fig. 3c). These results indicate that TLR5 signaling contributes to cytokine production induced by PHx.

Activation of NF-κB and STAT3 is well known as a major priming event during liver regeneration [9, 26]. NF-κB signaling was activated in mouse liver by PHx, as indicated by the phosphorylation and degradation of IκBα, and the phosphorylation of p65. The PHx-induced NF-κB activation in the liver was inhibited in *Tlr5*^−/−^ mice, as compared with WT mice, at 30 and 60 min after PHx (Fig. 3d). Both WT and *Tlr5*^−/−^ mice displayed increased hepatic STAT3 phosphorylation levels at 30 and 60 min after PHx, whereas *Tlr5*^−/−^ mice showed lower levels of phosphorylated STAT3 than WT mice (Fig. 3d). Together, these findings indicate that TLR5 contributes to the regulation of hepatocyte priming in liver regeneration after PHx.

### The TLR5 agonist CBLB502 enhances hepatocyte proliferation in mice after PHx

**Fig. 4:**
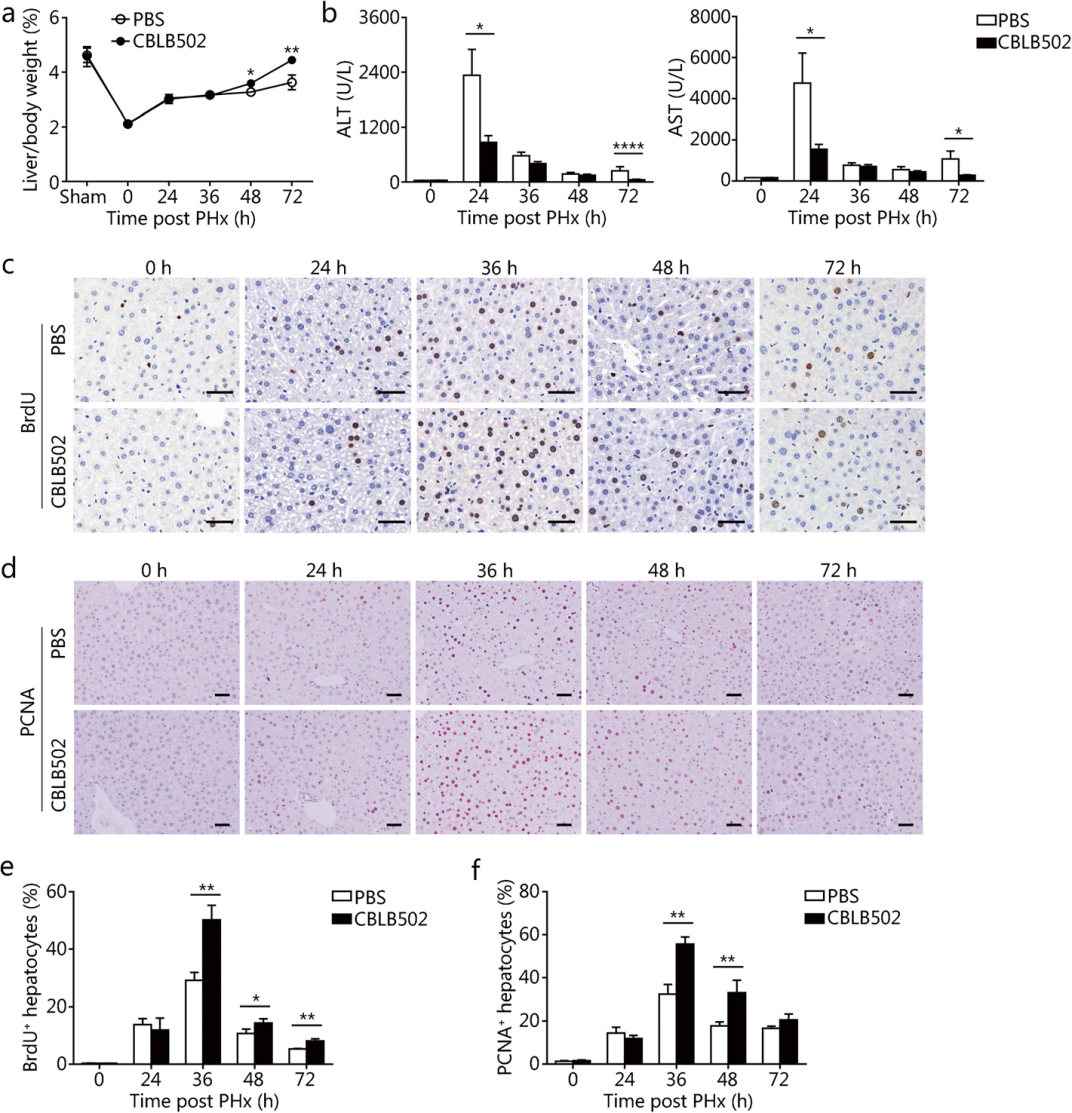
The TLR5 agonist CBLB502 enhances hepatocyte proliferation in mice after PHx. Liver to body weight ratio (**a**), and serum ALT and AST levels (**b**) in PBS and CBLB502 treated mice after PHx. Hepatocyte proliferation was measured with BrdU (**c**) and PCNA (**d**) immunohistochemistry staining. Percentages of BrdU-positive cells (**e**) and PCNA-positive cells (**f**) were counted at the indicated times after PHx. Results are expressed as the mean ± SEM. Data are derived from two or three independent experiments with similar results. *n* = 6–12. *P < 0.05, **P < 0.01

To further assess the role of the TLR5 pathway in liver regeneration, we investigated whether administration of CBLB502, a TLR5 agonist derived from *Salmonella* flagellin, might affect liver regeneration in mice after PHx. The liver/body weight ratio of CBLB502-pretreated mice was significantly higher than that of control mice during the first 72 h post-PHx, thus suggesting that activation of the TLR5 pathway is involved in the early recovery of liver mass after PHx (Fig. 4a). Accordingly, PHx-induced liver damage was reduced by CBLB502 administration at 24 and 72 h after PHx, as revealed by decreased serum transaminases (Fig. 4b). Hepatocyte proliferation was markedly enhanced in CBLB502 treated mice at 36, 48, and 72 h after hepatectomy by BrdU staining and 36 and 48 h by PCNA staining (Fig. 4c-f). Together, our results indicated that activation of TLR5 signaling by CBLB502 enhances hepatocyte proliferation in mice after PHx.

### Enhanced inflammatory response in CBLB502-pretreated mice after PHx

**Fig. 5:**
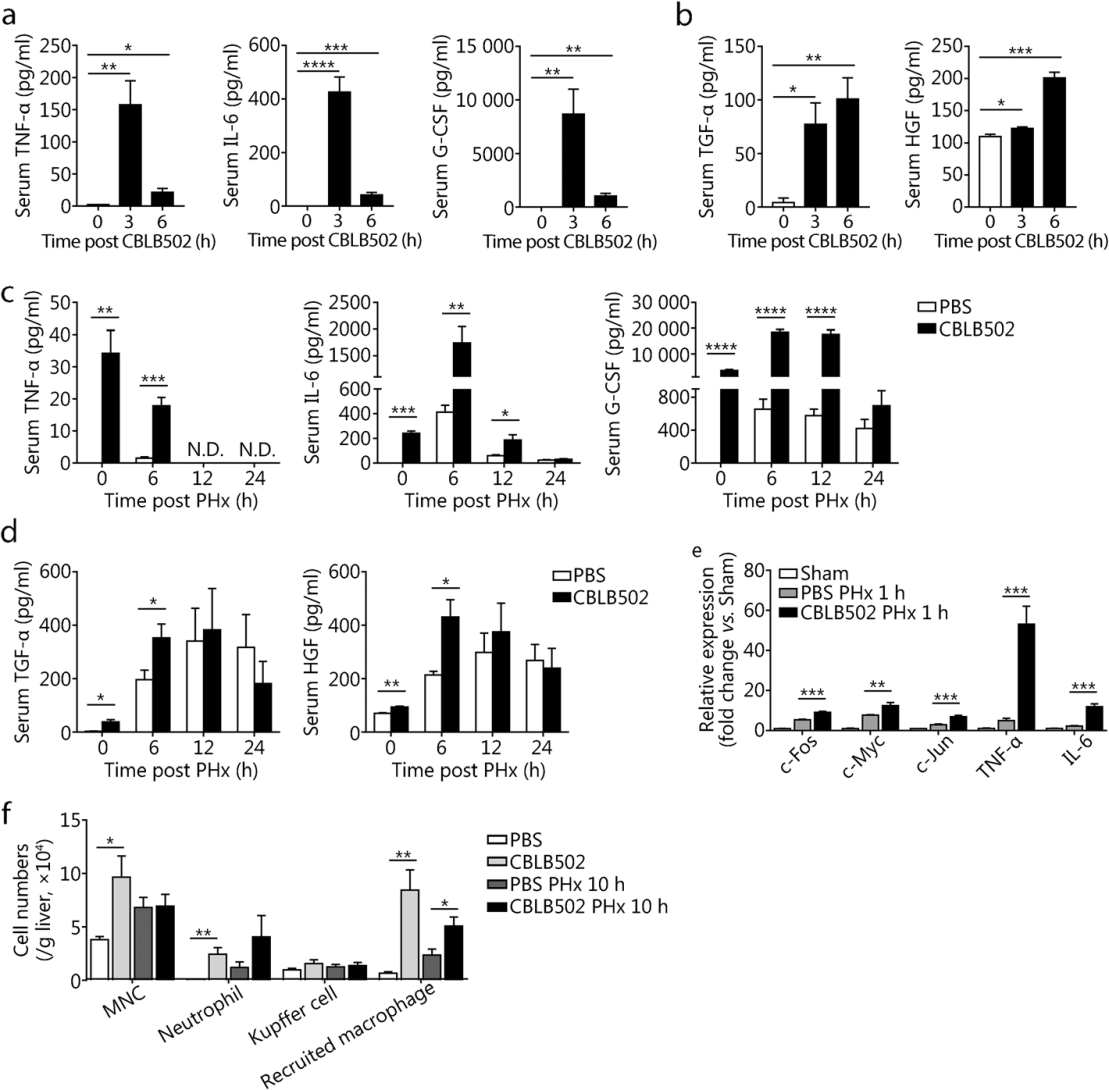
Enhanced inflammatory response in CBLB502-pretreated mice after PHx. Serum TNF-α, IL-6, and G-CSF (**a**), and TGF-α and HGF (**b**) in CBLB502 treated mice at the indicated times. Serum TNF-α, IL-6, and G-CSF (**c**), and TGF-α and HGF (**d**) were measured in regenerating PBS and CBLB502 treated mouse livers after PHx. **e** Relative mRNA levels of hepatic c-Fos, c-Myc, c-Jun, TNF-α, and IL-6 in PBS and CBLB502 treated mice after PHx. **f** Cell counts of liver MNCs, neutrophils, KCs, and recruited macrophages in PBS and CBLB502 treated mice after sham surgery or PHx. Panel **e** shows means ± SD. Data are representative of three independent experiments. Panels **a-d**, **f** show means ± SEM. Data are derived from two or three independent experiments with similar results. *n* = 3–7. N.D., not detected. *P < 0.05, **P < 0.01, ***P < 0.001, ****P < 0.0001

In line with previous reports [18], injection of CBLB502 in mice induced rapid increases in serum TNF-α, IL-6, and G-CSF (Fig. 5a). Interestingly, TGF-α and HGF, which play a vital role in hepatocyte proliferation [27, 28], were also significantly up-regulated in the serum at 3 and 6 h after CBLB502 injection (Fig. 5b). We next performed PHx 1 h after CBLB502 administration. Much higher levels of serum TNF-α, IL-6, G-CSF, TGF-α, and HGF were observed in CBLB502-pretreated mice than in control mice right before PHx (Fig. 5c-d). After PHx, the serum concentrations of these growth factors significantly increased in control mice, but the levels remained much lower than those in CBLB502-pretreated mice at 6 h after PHx (Fig. 5c-d). Moreover, the administration of CBLB502 significantly increased hepatic mRNA levels of c-Fos, c-Myc, c-Jun, TNF-α, and IL-6 at 1 h post-PHx (Fig. 5e), in agreement with the finding that TLR5 deficiency suppressed PHx-induced immediate early gene expression. We next examined whether CBLB502 might affect the hepatic recruitment of immune cells after PHx. As shown in Fig. 5f, the number of hepatic MNCs, neutrophils, and recruited macrophages was significantly higher in CBLB502 treated mice than in control mice before PHx, in line with previous reports that CBLB502 treatment induces recruitment of various types of immune cells into the liver [17, 18]. PHx also induced a significant increase in the total number of hepatic MNCs, as well as the number of recruited macrophages and neutrophils, and mice pretreated with CBLB502 showed a further increased number of these cells than mice pretreated with PBS. However, the number of KCs was not affected (Fig. 5f). Together, these data indicate that CBLB502 pretreatment increases the hepatic inflammatory response in mice after PHx.

### TLR5 signaling contributes to hepatic lipid accumulation induced by PHx

**Fig. 6:**
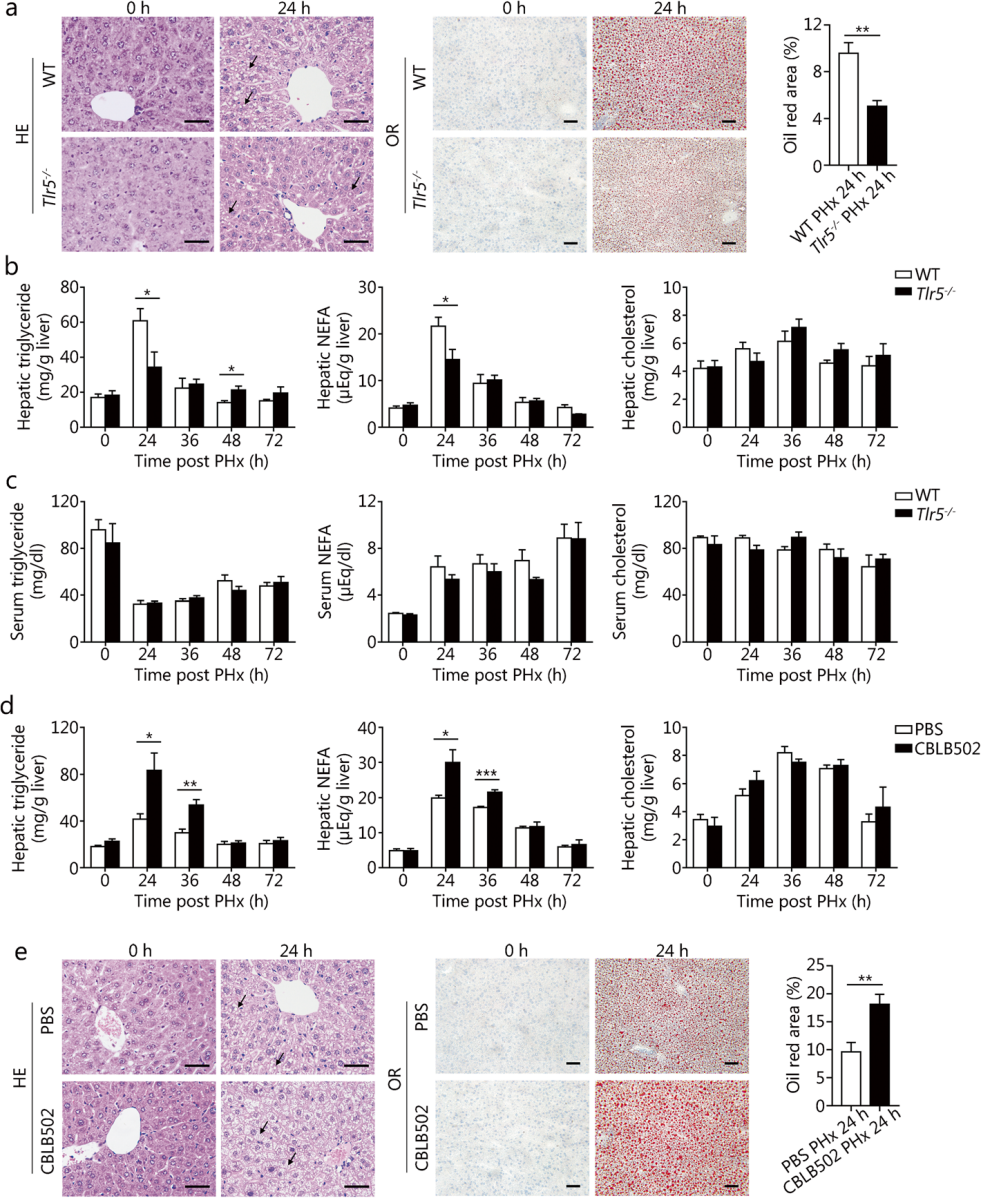
TLR5 signaling contributes to hepatic lipid accumulation induced by PHx. **a** H&E staining, Oil Red O staining, and quantification of positive areas of WT and *Tlr5*^−/−^ mouse livers after PHx. Triglyceride, NEFA, and cholesterol levels in liver extracts (**b**) and serum (**c**) were measured in WT and *Tlr5*^−/−^ mice at the indicated times after PHx. **d** Analysis of hepatic triglyceride, NEFA, and cholesterol levels in PBS and CBLB502 treated mice after PHx. **e** H&E staining, Oil Red O staining, and quantification of positive areas of PBS and CBLB502 treated mouse livers after PHx. Black arrows indicate hepatic lipids. Results are expressed as the mean ± SEM. Data are derived from two or three independent experiments with similar results. *n* = 5–7. *P < 0.05, **P < 0.01, ***P < 0.001

The transient hepatocellular fat accumulation following PHx is required for physiological liver regeneration [29, 30]. Histological analysis and Oil Red O staining showed a clear decrease in hepatic lipid accumulation in *Tlr5*^−/−^ mice at 24 h post-PHx (Fig. 6a). The levels of triglycerides, free fatty acids, and cholesterol in liver homogenates were comparable between WT and *Tlr5*^−/−^ mice under normal conditions (Fig. 6b). Significant increases in triglyceride and free fatty acid levels in liver homogenates were observed in both WT and *Tlr5*^−/−^ mice at 24 h after PHx, but the levels of triglycerides and free fatty acids in *Tlr5*^−/−^ mice were markedly lower than those in WT mice (Fig. 6b). TLR5 deficiency did not affect the levels of hepatic cholesterol and serum triglycerides, free fatty acids, and cholesterol, either before or after PHx (Fig. 6b-c). In agreement with these results, CBLB502 treatment increased hepatic triglyceride and free fatty acid accumulation at 24 and 36 h after PHx, but had no significant effect on the hepatic cholesterol content (Fig. 6d). Histological examination and Oil Red O staining of liver sections from CBLB502-treated and WT mice at different time points after PHx further confirmed these results (Fig. 6e). Collectively, these data indicate that the TLR5 signaling pathway is involved in the regulation of the hepatic lipid transient accumulation induced by PHx.

## Discussion

Previous studies have shown that activation of TLR5 signaling significantly affects the immune-privileged status of the liver and protects against Con A and Fas-agonistic antibody-induced liver injury [14–18]. However, the role of TLR5 signaling in liver regeneration has not been reported. In this study, we identified a role of TLR5 in liver regeneration, by using TLR5 knockout mice and mice treated with the TLR5 agonist CBLB502. We provided several lines of evidence suggesting that the activation of TLR5 signaling positively regulates liver regeneration via enhancing proinflammatory responses in the liver. First, the bacterial flagellin content increased in the serum and liver after PHx. Meanwhile, the expression of TLR5 in the liver was significantly up-regulated. Second, loss of TLR5 resulted in inhibition of PHx-induced liver regeneration, which was associated with attenuation of NF-κB and STAT3 activation, proinflammatory cytokine production, and immediate early gene expression. Third, activation of TLR5 signaling by the TLR5 agonist CBLB502 significantly promoted PHx-mediated hepatocyte proliferation, and was accompanied by enhanced production of proinflammatory cytokines and recruitment of macrophages and neutrophils in the liver. Altered microbiota composition is associated with elevated fecal bioactive flagellin and LPS levels, which may affect liver regeneration [31–35]. In the present study, we treated mice with gentamycin as previously described; this treatment results in comparable microbial compositions in the intestinal tract between *Tlr5*^−/−^ and WT mice [18], thereby eliminating the effects of intestinal pathogenic bacteria on PHx-induced liver regeneration in *Tlr5*^−/−^ mice. To our knowledge, this study is the first report that TLR5 is required for liver regeneration after PHx. CBLB502 is a pharmacologically optimized *Salmonella* flagellin derivative that is substantially less immunogenic than full-length flagellin but retains its TLR5-dependent NF-κB-inducing activity, and it is currently under development as an antitumor drug and medical radiation countermeasure [12, 17, 19]. Our studies have shown that the CBLB502-mediated protective effects against Con A-induced hepatitis and male reproductive system damage induced by ionizing radiation mainly rely on the TLR5 pathway [18], in line with the previous report that radioprotection by CBLB502 is indeed TLR5-dependent [12, 13]. Thus, our findings suggest that activation of TLR5 signaling may have the potential to improve liver regeneration when the liver is compromised.

It is well known that several cytokines such as TNF-α and IL-6 produced by nonparenchymal liver cells and transcription factors such as NF-κB and STAT3 are crucial for liver regeneration [2, 5]. After PHx, TLRs/MyD88-mediated pathways activate NF-κB pathways in nonparenchymal liver cells, induce TNF-α and IL-6 production, and trigger immediate early gene expression in hepatocytes [9]. In TLR5 knockout mice, initiation of liver regeneration is abated at the earlier time point, as evidenced by suppression of NF-κB and STAT3 activation, IL-6 and TNF-α production, and immediate early gene expression after PHx, thereby suggesting that TLR5 signaling is required for liver regeneration, particularly in the priming phase. This conclusion is supported by the finding that CBLB502 treatment enhanced hepatic c-Myc, c-Fos, c-Jun, IL-6, and TNF-α expression in mice in the early period after PHx. Although Burdelya et al. reported that hepatocytes were the effector cells that specifically and directly respond to CBLB502 [17], previous studies have shown very low levels of TLR5 expression on hepatocytes, as well as a fairly weak response of hepatocytes to CBLB502 in vivo [18, 36]; therefore, TLR5 signaling may regulate PHx-induced liver regeneration through nonparenchymal liver cells. The activation of NF-κB in KCs is almost universally accepted to be crucial for the initiation and intactness of liver regeneration after PHx [26, 37]. Importantly, KCs were found to express TLR5 in the present study, in agreement with previous reports [25]. Although the number of KCs in the liver and the expression of TLR5 on KCs were unaltered after PHx, the levels of bacterial flagellin in the serum and liver increased in PHx-treated mice. Thus, the activation of TLR5 signaling in KCs may be enhanced after PHx and contribute to liver regeneration. In addition, hepatic recruitment of macrophages and neutrophils, which have been shown to express TLR5 [18, 25], have been demonstrated to accelerate liver regeneration via the TNF/FasL/Fas or STAT3 pathway [38, 39]. After PHx, the expression of TLR5 on recruited macrophages and neutrophils was not altered, but their numbers significantly increased, suggesting that the up-regulation of hepatic TLR5 expression induced by PHx may be attributed to the influx of TLR5-positive immune cells. Studies by our group and others have shown that administration of CBLB502 rapidly induces the expression of numerous immunomodulatory factors, including TNF-α and IL-6, and massive recruitment of various types of immune cells, such as macrophages and neutrophils, in the liver of mice without PHx [17, 18]; this effect is further enhanced in the case of PHx. Therefore, the early and transient activation of hepatic TLR5 after PHx might possibly increase the inflammatory response during liver regeneration, through which TLR5 signaling promotes liver regeneration.

Except for TNF-α and IL-6, TLR5 signaling also affected TGF-α, HGF, and G-CSF expression after PHx. A marked decrease in systemic and liver local TGF-α, HGF, and G-CSF was detected in TLR5 knockout mice after PHx. In contrast, CBLB502 pretreatment significantly increased the expression of these proteins. TGF-α is an important growth factor in liver regeneration that directly stimulates DNA synthesis in hepatocytes [28], and HGF is produced in the liver by nonparenchymal cells and acts as a complete mitogen for efficient liver regeneration and repair [27, 40]. The important roles of these growth factors in liver regeneration have been extensively reported [37, 40]. These findings indicate that the TLR5 pathway also regulates the proliferation phase of liver regeneration through inducing hepatocyte proliferation-associated growth factors. The induction of G-CSF by CBLB502 plays an important role in the drug’s ability to protect mice against radiation injury [12]. Previous studies have also shown that G-CSF facilitates liver regeneration, through suppressing hepatic NK cells and increasing the migration of BM-derived progenitors to the liver [41, 42]. Our previous studies have shown that TLR5 signaling restrains T/NKT cells activation in the liver [18]. Thus, TLR5 signaling-induced facilitation of liver regeneration may also be achieved by immunoregulation of NK or/and NKT cells. It has been recognized that liver transiently accumulates hepatocellular fat after PHx, which is essential for hepatocyte proliferation [29, 30, 43]. TLR5 signaling significantly increases the levels of triglycerides and free fatty acids in the early regenerating liver, thus indicating that TLR5 signaling may contribute to lipid accumulation during early liver regeneration. These findings are worthy of further study in the future.

There are several limitations to this study. First, the analysis of the effect of the TLR5 pathway in liver regeneration focused on the priming phase and the proliferation phase after PHx, but it was unclear whether the TLR5 pathway would affect the termination phase in liver regeneration. Second, because the study was conducted in mice on a C57BL/6 background, more research is needed to confirm these results in other animal species, particularly primates. Additionally, our data indicate that TLR5 signaling contributes to hepatic lipid accumulation induced by PHx; however, the underlying mechanism still requires further study. Finally, our observations should be further verified with clinical samples before being extrapolated to humans.

## Conclusions

This work provides evidence that TLR5 activation contributes to the initial events in liver regeneration after PHx. Our findings enable a better understanding of the mechanisms of liver regeneration and suggest the potential of TLR5 agonist to promote liver regeneration.

## Supplementary Information

**Additional file 1: Table S1.** Antibodies used in flow cytometry and western blotting. **Table S2.** Primer sequences used in the study

## Data Availability

All materials are commercially available, and the datasets used and/or analyzed during the current study are available from the corresponding author on reasonable request.

## Abbreviations

ALT: Alanine transaminase
AST: Aspartate transaminase
BrdU: Bromodeoxyuridine
CBA: Cytometric bead array
Con A: Concanavalin A
H&E: Hematoxylin and eosin
IHC: Immunohistochemistry
IL-6: Interleukin-6
KCs: Kupffer cells
MNCs: Mononuclear cells
MyD88: Myeloid differentiation factor 88
PCNA: Proliferating cell nuclear antigen
PHx: Partial hepatectomy
SEM: Standard error of the mean
SPF: Specific pathogen-free
TLR5: Toll-like receptor 5
TLRs: Toll-like receptors
TNF-α: Tumor necrosis factor-α
WT: Wild-type
