# Supplementary material for: Toll-like receptor 5-mediated signaling enhances liver regeneration in mice
Source: Mil Med Res. 2021 Feb 23;8:16. doi: 10.1186/s40779-021-00309-4 (PMC7901072; doi:10.1186/s40779-021-00309-4)
Supplement: Supplementary file 1 — Additional file 1: Table S1. Antibodies used in flow cytometry and western blotting. Table S2. Primer sequences used in the study [file 40779_2021_309_MOESM1_ESM.docx]

**Supplementary material**

**Table S1. Antibodies used in flow cytometry and western blotting**

| **Antibodies** | **Source** | **Identifier** |
| --- | --- | --- |
| rabbit monoclonal anti- phospho-NF-κB p65 (Ser536) | Cell Signaling Technology | Cat#3033 |
| mouse monoclonal anti-IĸBα | Cell Signaling Technology | Cat# 4814 |
| rabbit monoclonal anti-ACTB | AB clonal | Cat# AC026 |
| rabbit monoclonal anti-NF-κB p65 | Cell Signaling Technology | Cat# 8242 |
| rabbit monoclonal anti-phospho-IκBα (Ser32) | Cell Signaling Technology | Cat# 2859 |
| mouse monoclonal anti-phospho-Stat3 (Tyr705) | Cell Signaling Technology | Cat# 9145 |
| mouse monoclonal anti-Stat3 | Cell Signaling Technology | Cat# 9139 |
| goat anti-mouse IgG | AB clonal | Cat# AS003 |
| goat anti-rabbit IgG | AB clonal | Cat# AS014 |
| anti-mouse F4/80-FITC | eBioscience | Cat# 11-480182 |
| anti-mouse CD45.2-PE-Cy7 | Biolegend | Cat# 109830 |
| anti-mouse Siglec F-APC | Biolegend | Cat# 155508 |
| anti-mouse Ly6G-eFluor 450 | eBioscience | Cat# 48-966882 |
| anti-mouse CD11b-BV605 | Biolegend | Cat# 101257 |
| anti-mouse TLR5-PE | Abcam | Cat# ab45119 |
| anti-mouse IgG2a | Abcam | Cat# ab91363 |

**Table S2. Primer sequences used in the study**

| Target | Type | Primer sense | Sequence (5’ → 3’) |
| --- | --- | --- | --- |
| *mActb* | qRT-PCR | F | AGAGGGAAATCGTGCGTGAC |
|  |  | R | CAATAGTGATGACCTGGCCGT |
| *mTnfa* | qRT-PCR | F | CTGAACTTCGGGGTGATCGG |
|  |  | R | GGCTTGTCACTCGAATTTTGAGA |
| *mIl6* | qRT-PCR | F | CTGCAAGAGACTTCCATCCAG |
|  |  | R | AGTGGTATAGACAGGTCTGTTGG |
| *mMyc* | qRT-PCR | F | CTGTCCATTCAAGCAGACGA |
|  |  | R | TCCAGCTCCTCCTCGAGTTA |
| *mJun* | qRT-PCR | F | TCCCCTATCGACATGGAGTC |
|  |  | R | TTTTGCGCTTTCAAGGTTTT |
| *mFos* | qRT-PCR | F | ATCCTTGGAGCCAGTCAAGA |
|  |  | R | ATGATGCCGGAAACAAGAAG |
| *mTgfa* | qRT-PCR | F | CACTCTGGGTACGTGGGTG |
|  |  | R | CACAGGTGATAATGAGGACAGC |
| *mHgf* | qRT-PCR | F | ATGTGGGGGACCAAACTTCTG |
|  |  | R | GGATGGCGACATGAAGCAG |
| *mTlr5* | qRT-PCR | F | ACCACACTTCAGCAGGATCA |
|  |  | R | ATCCAGGGAATCTGGGTGA |
